# Selection of Reference Genes for Quantitative Real-Time PCR in *Aquatica leii* (Coleoptera: Lampyridae) Under Five Different Experimental Conditions

**DOI:** 10.3389/fphys.2020.555233

**Published:** 2020-10-06

**Authors:** Xiao-Jie Yang, Hai-Long Zheng, Ying-Yang Liu, Hong-Wei Li, Yu-Hang Jiang, Lian-Bing Lin, Xian-Yu Deng, Qi-Lin Zhang

**Affiliations:** ^1^Faculty of Life Science and Technology, Kunming University of Science and Technology, Kunming, China; ^2^Engineering Research Center for Replacement Technology of Feed Antibiotics of Yunnan College, Kunming, China

**Keywords:** *Aquatica leii*, qPCR, reference gene, gene expression, stability evaluation

## Abstract

Aquatic fireflies are important indicators of the quality of freshwater environments and key models for research on insect adaptation to freshwater environments. For these investigations, gene expression analyses using quantitative real-time PCR are heavily dependent on reliable reference genes. In this study, based on a transcriptome assembly and annotation for the aquatic firefly *Aquatica leii* at the adult and larval stages, 10 candidate reference genes (α-*tubulin*, β-*tubulin*, β-*actin*, *EF1A*, *SDHA*, *UBQ*, *GST*, *GAPDH*, *RPS31*, and *RPL13A*) were identified for analyses of expression stability. Quantitative real-time PCR analyses for each candidate reference genes in *A. leii* was conducted for four developmental stages, four adult tissue types, two adult sexes, and two ecological stressors [adults exposed to five temperatures and larvae exposed to four concentrations of benzo(a)pyrene]. Results were evaluated by three independent algorithms (geNorm, NormFinder, and BestKeeper) and one comparative algorithm (RefFinder). The expression stability of candidate reference genes in *A. leii* differed under various conditions. Reference genes with the most stable expressions levels in different tissues, temperatures, sexes, developmental stages, and concentrations of benzo(a)pyrene were α-*tubulin*, *GST*, β-*actin*, β-*tubulin*, and α-*tubulin*, respectively. Furthermore, the optimal normalization factors (NFs) for the quantification of the expression levels of target genes by quantitative real-time PCR analyses of *A. leii* were identified for each experimental group. In particular, NF = 2 for different tissues (α-*tubulin* + β-*tubulin*), different sexes (β-*actin* + *EF1A*), and larvae exposed to different concentrations of benzo(a)pyrene (α-*tubulin* + *EF1A*); NF = 3 for developmental stages (*GST* + *GAPDH* + *SDHA*) and adults exposed to different temperatures (β-*tubulin* + *EFA* + *GST*). In addition, we surveyed the expression profiles of two target genes (*CYP3A* and *CSP8*) in larvae exposed to different concentrations of benzo(a)pyrene and in different adult tissues. The results further validated the reliability of the reference genes. The optimal reference genes for various experimental conditions identified in these analyses provide a useful tool for ecological studies of aquatic fireflies.

## Introduction

Quantitative real-time PCR (qPCR) is a key technique for the quantification of expression levels of target genes under different experimental condition in genetic and evolutionary research (Zhang Q. L. et al., 2016). Useful applications include studies of ecological adaptation, growth and development, comparative tissues analyses, and stress resistance (Feng et al., 2016; Tang et al., 2017; Zhang et al., 2017; Jia et al., 2019). Raw qPCR data must be normalized by suitable reference genes (RGs), to avoid expression differences among samples (Zhang Q. L. et al., 2016; Zhang et al., 2017). Ideally, expression levels of optimal RGs should not be influenced by experimental conditions (Scharlaken et al., 2008; Chapman and Waldenström, 2015). However, expression profiles of commonly used RGs are not universally stable across different experimental conditions, because these RGs participate in mRNA translation and cell metabolism (Zhang Q. L. et al., 2016; Zhang et al., 2017). For example, actin (*ACT*) and ribosomal RNA 18S (*18S*) are widely used as RGs in insects, but their expression is not stable among *Gynaephora* moths at different altitudes (Zhang et al., 2017). In addition, the optimal number (i.e., normalization factor, NF) of RG combinations for experiments should be determined to improve the accuracy of qPCR results by a multi-RG strategy (Bansal et al., 2016; Zhang et al., 2017). Therefore, the determination of suitable RGs and optimal NFs for different experiments is a prerequisite for the normalization of expression levels of target genes in qPCR analyses.

*Aquatica leii* belongs to the order Coleoptera, family Lampyridae, subfamily Luciolinae, genus *Aquatica* (Fu and Meyer-Rochow, 2012; Jiao et al., 2013) and is mainly distributed in the middle and lower reaches of the Yangtze River in mainland China. *A. leii* is the most widely studied aquatic firefly species. All adult fireflies are terrestrial, however, according to the habitats of larvae, species can be divided into aquatic, semi-aquatic, and terrestrial types (Fu, 2005; Fu et al., 2012). *A. leii* is an important insect with a delight to watch and an ecological indicator owing to its flashing behavior and high sensitivity to deteriorating water quality caused by water pollutants (Fu, 2005; Zhang et al., 2019).

In recent years, increasing water pollution, artificial light source interference, commercial capture, climate change, and other human activities, have dramatically decreased the population size of aquatic fireflies (Fu, 2005; Zhang et al., 2019). Cold hardiness resulting from climate change is a crucial strategy for survival in insects. Thus, temperature largely determines the distribution, abundance, and rates of development of insects (Jing and Kang, 2002). Therefore, to protect aquatic firefly resources, it is essential to understand the mechanisms underlying ecological adaptation (e.g., water pollutants and temperature) at gene expression levels. Furthermore, gene expression across firefly lineage and its tissue specificity are important indicators for the study of the mechanisms underlying freshwater adaptation (Zhang et al., 2020). These genes can be identified via intraspecific gene expression difference comparisons, such as different developmental stages (terrestrial adults vs. aquatic larvae), sexes and tissues, as well as interspecific comparisons (e.g., aquatic vs. terrestrial fireflies) (Zhang et al., 2020). Therefore, it is important to identify suitable RGs for the calibration of target gene expression under different experimental conditions (e.g., developmental stages, tissues, sex, temperature, and particular water pollutants) in aquatic fireflies.

In this study, expression of 10 candidate RGs (Table 1) widely used for qPCR analyses of insects is not universally stable among different experimental conditions (Liu et al., 2014; Jia et al., 2019). Expression levels of these 10 candidate RGs were quantified in *A. leii* in four developmental stages, four adult tissues types, different sexes, and two ecological stressors (temperatures and water pollutants). Subsequently, the expression stability of RGs by qPCR was evaluated using the following four algorithms: geNorm, NormFinder, BestKeeper, and RefFinder. The optimal NFs for qPCR in five different experimental groups were determined using the geNorm algorithm. Finally, two target genes, *CYP3A* encoding cytochrome P450 3A and *CSP8* encoding chemosensory protein 8, which have important detoxifying and olfactory functions, were chosen for validation.

**TABLE 1:** Details of candidate RGs.

Gene	Gene name	Unigene id	Primer sequences (5′-3′)	Amplicon size (bp)	Slope	Correlation coefficient/*R*^2^	Amplification efficiency (%)
α-*tubulin*	Alpha tubulin	c102349.graph_c0	F: CGTTTTGTCGGGTACATAT R: AACACGCATTTCCTATTTGA	138	−3.317	0.998	100.21
β-*tubulin*	Beta tubulin	c91143.graph_c0	F: ACCTTACAATGCCACTCTT R: CCGTATGTTGGTGTCGTA	126	−3.390	0.997	97.24
β-*actin*	Beta actin	c130645.graph_c0	F: TTCTTCCATAGCGGTGAA R: CCAGCAGTTGTTCTTGAC	147	−3.290	0.999	101.35
*EF1A*	Elongation factor 1-alpha	c133684.graph_c0	F: TGAAACCTTTGCTGAATACC R: GTCCTTCTTGTCAACATTCTT	101	−3.382	0.997	97.55
*SDHA*	Succinate dehydrogenase	c146902.graph_c0	F: TGATTGACGCTCTTGAGT R: AAGGTGTGTTTCATCCATTT	145	−3.310	0.990	100.50
*UBQ*	Ubiquitin	c150294.graph_c0	F: GCTCTTCCGATCTCACAA R: AATGCTCTTATGACTGGTTTC	122	−3.428	0.999	95.76
*GST*	Glutathione S-transferase	c158765.graph_c0	F: CGGGTGGGTATAGAGTTT R: ACTGAACGGAATAGAACCT	129	−3.354	0.998	98.68
*GAPDH*	Glyceraldehyde-3-phosphate dehydrogenase	c123644.graph_c0	F: CATACGAATAGTCCATAACGATT R: AGGGTGTTGCTTCTCATT	140	−3.395	0.998	97.04
*RPS31*	Ribosomal protein S31	c132038.graph_c1	F: TATCGGAAGTTGGTGCTT R: AACGGTAACAGGACTCAA	135	−3.266	0.998	102.41
*RPL13A*	Ribosomal protein L13A	c116605.graph_c0	F: TTAGAGCACCATCCAGAAT R: TTCATAACACTTCAGCCTTC	102	−3.292	0.996	101.27

## Materials and Methods

### Animals

*Aquatica leii* was collected from the Culture Center of Fireflies, Ganzhou city, Jiangxi province, China. Before collecting or treating *A. leii*, larvae were maintained in a plastic box (40 cm × 30 cm × 10 cm) at 24−27°C under an 8-h light/16-h dark photoperiod, with oxygenators, heating rods (to increase the water temperature) and 5−6 cm of water (changed every 5 days) (detailed processes for the cultivation of fireflies are described in Zhang et al., 2020). *A. leii* adults were reared at 90 ± 5% relative humidity, under the same temperature and photoperiod cycle used for larvae. The expression of the reference genes was studied in animals and tissues which represented five experimental groups. First, different developmental stages; second, various types of adult tissues; third, adult sexes; fourth, adults exposed to different temperatures; and fifth, larvae exposed to a water contaminant. (1) Different developmental stage were collected from adult *A. leii*, such as 200 eggs, 20 larvae (4−6 instar mixture), 5 pupae, and 10 adults (a mixture of males and females). (2) Different tissues were dissected from adult *A. leii*, such as 30 heads, 30 thorax, 20 abdomen, and 100 antenna. (3) Ten individuals from each of adult female and male *A. leii* were collected. (4) Adult *A. leii* exposed to various temperatures for 2 h were collected, 10 individuals per different temperature, including 15°C, 20°C, 25°C, 30°C, and 35°C. (5) Due to ecological deterioration and intensifying human activity, polycyclic aromatic hydrocarbons (PAHs) can be easily detected in freshwater environments. Benzo(a)pyrene (BaP) is a PAH known as one of the most potent environmental carcinogens, and intensifying human activity is the main cause of its release into rivers and lakes (Xiao et al., 2018). Animals at the 4−6 larval instar stages were exposed to different concentrations of BaP for 24 h were collected as water contaminant-exposed groups, including control, 0.1, 0.01, and 0.001 mg/L, 20 individuals per concentration. Animals were collected three separate times, resulting in three biological replicates for each experimental group. All collected samples were immediately frozen in liquid nitrogen after labeling and stored at −80°C at the Faculty of Life Science and Technology, Kunming University of Science and Technology, Kunming, China.

### RNA Extraction and cDNA Synthesis

Total RNA was extracted from each sample using TRIzol reagent (Ambion, Austin, TX, United States) according to the manufacturer’s instructions. RNase-free DNase (Qiagen, Hilden, Germany) was used to remove residual genomic DNA, following the manufacturer’s instructions. Three biological replicates were performed per sample. RNA quality and quantity were measured using a Nanodrop 1000 spectrophotometer (Thermo Fisher Scientific, Waltham, MA, United States), and RNA integrity was confirmed by 1.5% agarose gel electrophoresis. Only qualified RNA with an OD_260_/OD_280_ ratio between 1.8 and 2.0 and a concentration exceeding 300 ng/μL was retained for downstream analyses. Single-stranded cDNA was synthesized in a total volume of 20 μL, containing 4 μL of 5 × PrimeScript RT Master Mix (including oligo dT primers; TaKaRa, Kusatsu, Japan), 1 μL of total RNA (200 ng/μL), and 15 μL of RNase-free ddH_2_O at 37°C for 15 min and 85°C for 5 s, according to the manufacturer’s protocol with modifications. cDNA (100 ng/μL) was diluted with RNase-free water and used for subsequent experiments.

### Design and Evaluation of Primers for Each Gene

According to previous studies (Liu et al., 2014; Nagy et al., 2017; Zhang et al., 2017; Jia et al., 2019), α-*tubulin*, β-*tubulin*, β-*actin*, *EF1A*, *SDHA*, *UBQ*, *GST*, *GAPDH*, *RPS31*, and *RPL13A* were identified as candidate RGs. These genes were identified from mixed assembled transcriptome (plus annotation) results for larval and adult *A. leii* (Zhang et al., 2020); original clean reads used for the assembly were downloaded from the NCBI Sequence Read Archive database, under accession no. PRJNA555399. All specific primers were designed using Beacon Designer 7.0. The specificity of the amplification product for each primer was checked by the appearance of a single band at the targeted length by 2% agarose gel electrophoresis. Detailed information for qPCR primers is presented in Table 1. Furthermore, all gene-specific amplified PCR products were confirmed by sequencing. The qPCR amplification efficiency (*E*) and correlation coefficient (*R*^2^) of each primer were calculated using a standard curve generated from a 10-fold dilution series of mixed cDNA samples at five dilutions (1/10, 1/100, 1/1000, 1/10000, and 1/100000). The corresponding qPCR amplification efficiency (*E*) was calculated according to the following formula: *E*(%) = (10^(−1/slope)^− 1)× 100 (Radoniæ et al., 2004; Zhang Q. L. et al., 2016).

### Quantitative RT-PCR

Reactions were prepared in a total volume of 10 μL, including 1 μL of diluted cDNA (100 ng/μL), 5 μL of 2 × SYBR Premix ExTaq II (TaKaRa, Kusatsu, Japan), and 0.5 μL of each of the forward and reverse primers (10 ng/μL). RNase-free sterile water was added to obtain the final volume. The PCR program was as follows: 95°C for 35 s, 40 cycles of 95°C for 5 s, 55°C for 30 s, and 72°C for 30 s. A melting curve analysis was performed to confirm the amplification specificity for each reaction. A reaction solution without cDNA template was used as negative control to confirm template-specific amplification. PCRs were conducted for three biological replicates, and the detection of each gene was performed in an independent sample with three technical replicates. All qPCRs were conducted using an ABI 7300 real-time PCR system (Applied Biosystems, Waltham, MA, United States).

### Determining the Expression Stability of Candidate RGs

Data for each of the five experimental groups were analyzed independently. Average Ct values for each candidate RG were calculated based on data for three biological and three technical replicates (*n* = 9) in each sample. BestKeeper (an Excel-based algorithm^1^) was employed to evaluate the expression stability of candidate RGs according to the correlation coefficient (*r*), standard deviation (*SD*), and coefficient of variation (*CV*) generated by pairwise comparison between candidate RGs. Smaller *SD* and *CV*-values indicated more stable expression levels of RGs (Pfaffl et al., 2004). geNorm^2^ sorted expression stabilities according to the *M*-values of candidate RGs (Vandesompele et al., 2002). A lower *M*-value indicated higher expression stability (Vandesompele et al., 2002). geNorm also calculates pairwise variation (*V*) between each RG and all other RGs to determine the NFs of RGs (Vandesompele et al., 2002). The value of *V*_n/(n+1)_ for two sequential NFs was used to determine the optimal number of RGs required for normalization. Values below a threshold of 0.15 suggest that no additional RGs are needed for normalization (Vandesompele et al., 2002). NormFinder^3^ assessed the expression stability of candidate RGs based on the stability value (*SV*) and *SD* of Ct values. A lower *SV* in NormFinder indicated more stable expression levels of RGs (Andersen et al., 2004). Finally, these candidate RGs were ranked using the web-based analytical tool RefFinder^4^, a comprehensive ranking tool that integrates different programs (Silver et al., 2006).

### Validation of RGs

*CYP3A*, a detoxifying protein involved in the response to oxidative stress in insects, is up-regulated in *A. leii* in response to BaP exposure (Zhang et al., 2019). Chemosensory proteins, including olfaction-related genes in insect antennae, play important roles in olfaction (Zhang L. W. et al., 2016; Wang et al., 2017). Previous studies have reported the antenna-specific expression of *CSPs* in insects (Zhao et al., 2018). To confirm the reliability of the selected RGs, the expression profiles of the target gene *CYP3A* and *CSP8* were determined in different concentrations of BaP and adult tissues, respectively, and independently normalized with the two most stable RGs as well as the least stable RGs. The PCR system and program were the same as those for the qPCR analysis of RGs. In qPCR analyses, three technical and three biological replicates were conducted for each treatment. Normalization of expression levels of target genes was performed according to the 2^–ΔΔCT^ method (Livak and Schmittgen, 2001). Differences in target gene expression levels among concentrations and tissues were valuated by one-way ANOVA plus Bonferroni *post hoc* tests implemented in IBM SPSS 22.0.

## Results

### Expression Profiles of Candidate RGs

**FIGURE 1:**
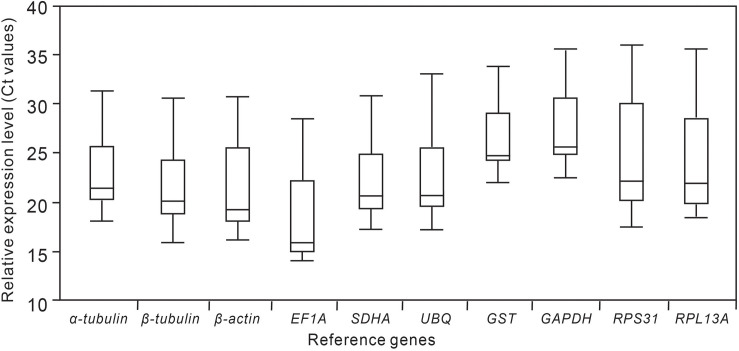
Expression levels of candidate reference genes in different samples. The expression levels are presented in terms of the raw cycle threshold number (Ct values) of the candidate reference genes in different samples. The line in the box indicates the median. The box ranges between the 25th and the 75th percentiles, and the maximum and minimum values are represented by upper and lower caps.

The specificity of gene amplification of all candidate RGs was confirmed by a single band with expected molecular mass using 1.5% agarose (Supplementary Figure 1). A standard curve was generated for each gene based on ten-fold serial dilution of the pooled cDNA at five dilutions. The amplification efficiencies of all candidate RGs were 95.76−102.41%, with correlation coefficients (*R*^2^) exceeding 0.995 (Table 1). The Ct value was the only parameter used to quantify the expression level of candidate RGs. Changes in Ct values of each candidate RG under five different experimental conditions are shown in Figure 1 and Supplementary Table 1. There was a wide range of expression levels and high variation across samples in expression patterns for the ten candidate RGs in each experimental group (Supplementary Figure 2). *EF1A* had the lowest Ct values, while *GAPDH* had the highest Ct values; the median Ct values for the 10 candidate RGs ranged from 15 to 25. Variance in Ct values decreased in the following order: *GST* > α-*tubulin* > β-*tubulin* > *SDHA* > *GAPDH* > *UBQ* > *EF1A* > β-*actin* > *RPL13A* > *RPL31*.

### Expression Stability of Candidate RGs Determined Using BestKeeper

In different tissue types of adult *A. leii*, the *SD* values for α-*tubulin* and β-*tubulin* were less than 1, and values for other candidate RGs exceeded 1. α-*Tubulin* and β-*tubulin* showed high expression stability. In adult *A. leii* exposed to different temperatures, only the *SD* value of *GST* was less than 1; suggesting that *GST* has the most stable expression. Among other candidate RGs, *RPS31* expression was most unstable. In adults of different sexes, the *SD* values of *UBQ* and *GAPDH* exceeded 1, and showed the least stable expression. β-*Tubulin*, *EF1A*, and β-*actin* had the most stable expression, followed by *RPL13A*, α-*tubulin*, *GST*, *SDHA*, and *RPS31*. At different developmental stages, the expression of *GAPDH* was most stable. In larvae exposed to different concentrations of BaP, the *SD* values for all 10 candidate RGs were below 1, and the expression stability of these candidates could be ordered from high to low as follows: α-*tubulin*, *EF1A*, *RPS31*, β-*tubulin*, *GST*, β-*actin*, *UBQ*, *SDHA*, *GAPDH*, and *RPL13A* (Table 2 and Supplementary Table 2).

**TABLE 2:** Expression stability ranking of candidate reference genes in *Aquatica leii* under five different experimental groups.

Groups	Reference genes	BestKeeper	geNorm	NormFinder
Tissues	α-*tubulin*	1	1	1
	β-*tubulin*	2	1	2
	β-*actin*	10	8	9
	*EF1A*	4	6	7
	*SDHA*	7	4	6
	*UBQ*	6	5	5
	*GST*	3	3	4
	*GAPDH*	9	9	10
	*RPS31*	5	2	3
	*RPL13A*	8	7	8
Temperatures	α-*tubulin*	2	4	5
	β-*tubulin*	3	5	6
	β-*actin*	8	3	4
	*EF1A*	5	6	8
	*SDHA*	4	2	3
	*UBQ*	7	7	7
	*GST*	1	1	1
	*GAPDH*	6	1	2
	*RPS31*	10	9	10
	*RPL13A*	9	8	9
Sexs	α-*tubulin*	5	4	5
	β-*tubulin*	1	3	3
	β-*actin*	3	1	1
	*EF1A*	2	2	2
	*SDHA*	7	5	6
	*UBQ*	9	9	10
	*GST*	6	7	9
	*GAPDH*	10	8	7
	*RPS31*	8	1	4
	*RPL13A*	4	6	8
Developmental	α-*tubulin*	8	4	5
stages	β-*tubulin*	3	1	1
	β-*actin*	9	3	4
	*EF1A*	4	2	2
	*SDHA*	2	5	6
	*UBQ*	10	8	9
	*GST*	5	1	3
	*GAPDH*	1	6	7
	*RPS31*	6	7	8
	*RPL13A*	7	9	10
Benzo(a)pyrene	α-*tubulin*	1	1	2
	β-*tubulin*	4	3	4
	β-*actin*	6	8	8
	*EF1A*	2	1	3
	*SDHA*	8	4	5
	*UBQ*	7	7	9
	*GST*	5	5	7
	*GAPDH*	9	6	6
	*RPS31*	3	2	1
	RPL13A	10	9	10

### Expression Stability of Candidate RGs Determined Using geNorm

In different adult tissues of *A. leii*, the three candidate RGs with the highest expression stabilities were α-*tubulin*, β-*tubulin*, and *RPS31*. The three candidate RGs with the most unstable expression levels were *GAPDH*, β-*actin*, and *RPL13A*. In adults exposed to different temperatures, the three candidate RGs with the highest expression stabilities were *GST*, *GAPDH*, and *SDHA*, and the three candidate RGs with the most unstable expression levels were *RPS31*, *RPL13A*, and *UBQ*. In adults of different sexes, the three candidate RGs with the highest expression stabilities were β-*actin*, *RPS31*, and *EF1A*, and three candidates with the worst expression stabilities were *UBQ*, *GAPDH*, and *GST*. At different developmental stages, the three most stable candidate RGs were β-*tubulin*, *GST*, *EF1A*, *RPL13A*, and *UBQ*, while *RPS31* showed the most unstable expression. In larvae exposed to different concentrations of BaP, the three most stable candidates were α-*tubulin*, *EF1A*, and *RPS31*, while the most unstable candidates were *RPL13A*, β-*actin*, and *UBQ* (Table 2 and Supplementary Table 3).

### Expression Stability of Candidate RGs Determined Using NormFinder

The NormFinder analysis showed that the expression stability of candidate RGs in different adult tissues was in the following order: α-*tubulin* > β-*tubulin* > *RPS31* > *GST* > *UBQ* > *SDHA* > *EF1A* > *RPL13A* > β-*actin* > *GAPDH*. In adults exposed to different temperatures, the expression stability of candidate RGs decreased in the following order: *GST* > *GAPDH* > *SDHA* > β-*actin* > α-*tubulin* > β-*tubulin* > *UBQ* > *EF1A* > *RPL13A* > *RPS31*. In adults of different sexes, the expression stabilities of candidate RGs ranked as follows: β-*actin* > *EF1A* > β-*tubulin* > *RPS31* > α-*tubulin* > *SDHA* > *GAPDH* > *RPL13A* > *GST* > *UBQ*. At different developmental stages, the expression stabilities of candidate RGs decreased in the following order: β-*tubulin* > *EF1A* > *GST* > β-*actin* > α-*tubulin* > *SDHA* > *GAPDH* > *RPS31* > *UBQ* > *RPL13A*. In larvae exposed to different concentrations of BaP, the expression stabilities of candidate RGs decreased in the following order: *RPS31* > α-*tubulin* > *EF1A* > β-*tubulin* > *SDHA* > *GAPDH* > *GST* > β-*actin* > *UBQ* > *RPL13A* (Table 2 and Supplementary Table 4).

### Expression Stability of Candidate RGs Determined Using RefFinder

The expression stabilities of candidate RGs in different adult tissues decreased in the following order: α-*tubulin* > β-*tubulin* > *RPS31* > *GST* > *UBQ* > *SDHA* > *EF1A* > *RPL13A* > β-*actin* > *GAPDH*. In adults exposed to different temperatures, the expression stabilities of candidate RGs decreased in the following order: *GST* > *GAPDH* > *SDHA* > α-*tubulin* > β-*tubuli*n > β-*actin* > *EF1A* > *UBQ* > *RPL13A* > *RPS31*. In adults of different sexes, the expression stabilities of candidate RGs decreased in the following order: β-*actin* > *EF1A* > β-*tubulin* > *RPS31* > α-*tubulin* > *SDHA* > *RPL13A* > *GST* > *GAPDH* > *UBQ*. At different developmental stages, the expression stabilities of candidate RGs decreased in the following order: β-*tubulin* > *EF1A* > *GST* > *SDHA* > *GAPDH* > β-*actin* > α-*tubulin* > *RPS31* > *RPL13A* > *UBQ*. In larvae exposed to different concentrations of BaP, the expression stabilities of candidate RGs decreased in the following order: α-*tubulin* > *EF1A* > *RPS31* > β-*tubulin* > *SDHA* > *GST* > *GAPDH* > β-*actin* > *UBQ* > *RPL13A* (Table 3).

**TABLE 3:** Expression stability of candidate reference genes in *Aquatica leii* under five different experimental groups calculated by RefFinder.

Genes	Tissues		Temperatures		Sexs		Development stages		Benzo(a)pyrene	
	Rank	GM	Rank	GM	Rank	GM	Rank	GM	Rank	GM
α-*tubulin*	1	2.93	4	4.92	5	5.87	7	6.37	1	1.74
β-*tubulin*	2	3.56	5	5.13	3	2.62	1	2.24	4	3.89
β-*actin*	9	11.16	6	6.27	1	1.92	6	6.11	8	8.06
*EF1A*	7	6.53	7	6.41	2	2.14	2	3.52	2	2.15
*SDHA*	6	6.34	3	3.87	6	6.08	4	5.78	5	6.37
*UBQ*	5	5.45	8	7.39	10	9.86	10	13.15	9	8.48
*GST*	4	4.62	1	1.82	8	7.44	3	3.61	6	6.81
*GAPDH*	10	12.08	2	2.76	9	8.73	5	5.92	7	7.59
*RPS31*	3	3.89	10	9.27	4	3.66	8	9.73	3	2.56
*RPL13A*	8	7.37	9	8.11	7	7.26	9	12.38	10	9.92

*GM indicates geometric mean.*

### Determination of Optimal NFs

**FIGURE 2:**
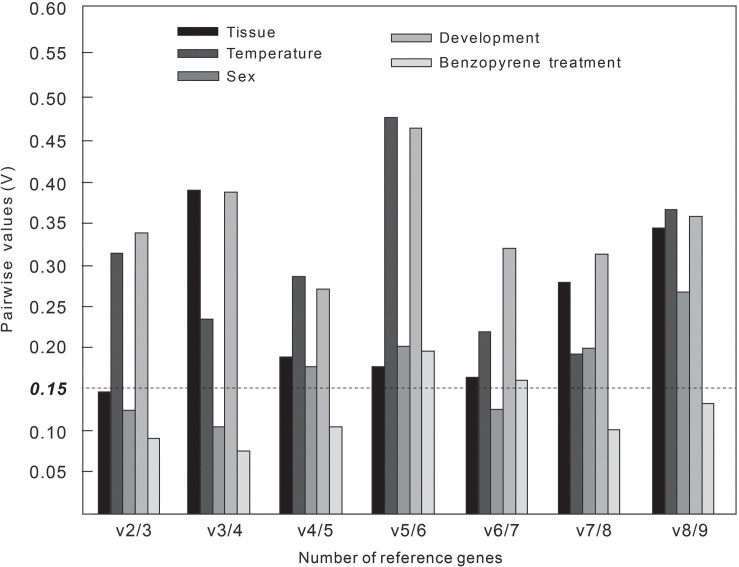
Number of optimal combinations of reference genes under five different experimental groups.

If *V*-value is initially lower than the default value 0.15 in all pairwise variation, or *V*-value is lowest when all the *V*-values are more than 0.15, the number of the corresponding gene pairings will be good enough for normalization (Zhang et al., 2017). The *V*_2/3_ value was first lower than 0.15 in all pairwise variation in adult tissues, sexes, and larval BaP concentrations (Figure 2), indicating that the optimal number of RGs for normalization was two for the above three experimental groups, and the combinations were α-*tubulin* + β-*tubulin*, β-*actin* + *EF1A*, and α-*tubulin* + *EF1A*, respectively (Table 4). At different developmental stages and temperatures, all *V*_n/(n+1)_ values exceeded 0.15, and the lowest value was more than *V*_4/5_ and *V*_7/8_, respectively. According to the geNorm recommendation, the optimal number of NFs was three, and the optimal combinations of RGs were *GST* + GA*P*DH + *SDHA* and β-*tubulin* + *EFA* + *GST*, respectively.

**TABLE 4:** Optimal combination of reference genes in *Aquatica leii* under five different experimental groups.

Groups	The most stably expressed gene	Optimal combination of reference genes
Tissue	α-*tubulin*	α-*tubulin* +β-*tubulin*
Temperature	*GST*	*GST*+*GAPDH*+*SDHA*
Sex	β-*actin*	β-*actin*+*EF1A*
Developmental stages	β-*tubulin*	β-*tubulin*+*EF1A*+*GST*
Different dose of benzo(a)pyrene	α-*tubulin*	α-*tubulin*+*EF1A*

### Validation of RGs

**FIGURE 3:**
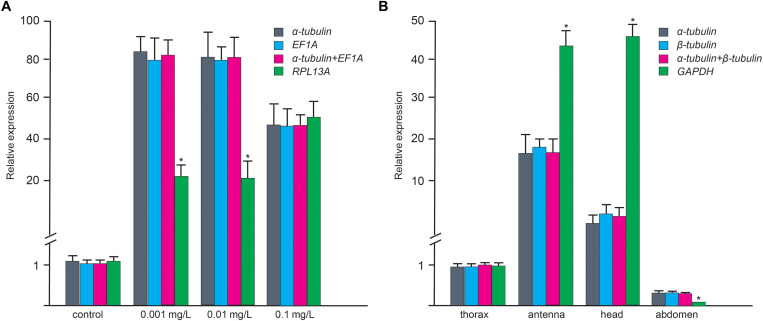
Validation of selected reference genes in larval *A. leii* under different BaP concentrations **(A)** and adult tissues **(B)** in *A. leii*. Relative expression level of the target genes (*CYP3A* and *CSP8*) in different samples using different normalization factors (the most and least stable genes). Asterisks indicate significant differences in the expression levels of the target genes.

*CYP3A* and *CSP8* were selected as target genes to validate the applicability of the selected RGs. Under the most stable RGs (α-*tubulin* and/or *EF1A*) at different BaP concentrations, the expression patterns of *CYP3A* were consistent with down-regulation at low BaP concentration period and up-regulation at higher concentration period. This indicates that we have shown a reliable expression pattern of *CYP3A* using the most stable RGs screened in this study [higher expression level of *CYP3A* under higher BaP concentrations (Zhang et al., 2019)]. Nevertheless, when the most unstable RG (*RPL13A*) was employed to normalize expression levels of target genes, *CYP3A* failed to present a consistent expression pattern (Figure 3). In different adult tissues, when the most stable RGs (α-*tubulin* and/or β-*tubulin*) were used as normalization factors, the expression of the target gene *CSP8* was highest in the antenna. However, when the most unstable RG (*GAPDH*) was employed, the expression pattern of *CSP8* significantly differed with normalization by α-*tubulin* and α-*tubulin* + β-*tubulin* (Figure 3). It is generally true that the accurate expression of target genes could be obtained, when the most stable RGs are employed as normalization factors.

## Discussion

In this study, the results obtained by different independent algorithms were highly consistent. For example, the stability ranking of candidate RGs in geNorm was highly similar to that of NormFinder. However, there were also differences among algorithms with respect to the expression stability of candidate RGs, as reported in many similar studies (e.g., Ibanez and Tamborindeguy, 2016; Koramutla et al., 2016; Zhang et al., 2017). A comparison of the three algorithms used here showed that BestKeeper exhibited the most discrepancies, consistent with previous reports (Lu et al., 2015; Zhang et al., 2017). This result indicated that it is essential to select optimal RGs under specific conditions using multiple independent algorithms.

In the current study, we evaluated common genes used for normalization. α-*Tubulin* and β-*tubulin* showed high expression stability in different adult tissues and in larvae exposed to different BaP concentrations in *A. leii*. Even β-*tubulin* retained a highly stable expression level in different adult sexes and developmental stages, indicating that β-*tubulin* is a suitable RG for *A. leii* under various experimental conditions. Therefore, when several limitations (e.g., limited samples, time, and funds) prevented experiments that screened the most suitable RGs, α-*tubulin* and β-*tubulin* can be considered as optimal RGs for qPCR analyses of target genes in fireflies. β-*Actin*, a traditional insect RG, showed high expression stability only in the sex group and showed either low or the lowest expression stability in the other four experimental conditions evaluated. Similar results were reported in investigations of RGs in insects belonging to Hemiptera and Lepidoptera (Zhang et al., 2017; Jia et al., 2019). Accordingly, traditional and widely used RGs may not be stably expressed in many experimental conditions and thus should be used cautiously for qPCR analyses. *EF1A* showed extreme expression stability between sexes, developmental stages, and larvae exposed to different concentrations of BaP in *A. leii*. This was consistent with the results of RG selection for qPCR analyses of grassland caterpillars (genus *Gynaephora*) along an altitudinal gradient (Zhang et al., 2017). However, the expression stability of *EF1A* was low among different adult tissues and temperature treatments. These results provided a useful insight into the appropriate conditions for the application of *EF1A* in *A. leii*. *GST* showed the highest expression stability in *A. leii* adults exposed to different temperatures; thus, this gene could be considered a suitable RG for qPCR analyses of the temperature response of *A. leii*. *RPS31* and *RPL13A* are ribosomal protein-coding genes. Several studies have reported that these two candidate RGs are stably expressed in *Lygus pratensis*, and participate in protein translation and synthesis (Jia et al., 2019). Our results showed that the expression stability of *RPS31* was higher than that of *RPL13A* in five experimental conditions in *A. leii*. This indicates that candidate RGs with similar biological functions exhibit differences in expression stability. Both *SDHA* and *GAPDH* showed high expression stability in several experimental conditions in *A. leii*; thus, these two genes should also be considered candidates for studies of other firefly groups. *UBQ* showed moderate or low expression stability in all five experimental conditions evaluated; therefore, *UBQ* can be excluded from candidate RGs in the future.

Many studies have proposed that multiple RGs should be used in combination to improve normalization (i.e., biased normalization can be removed via mutiple-RG correction) (Kumar et al., 2012; Fu et al., 2013; Dzaki and Azzam, 2018). The optimal numbers of NFs were seven and four for *A. leii* at different temperatures and developmental stages, respectively. However, owing to limited experimental resources and time, it is often unrealistic to use many RGs in combination in practical applications. Moreover, an excessive number of RGs could introduce more uncertainty and error, decreasing the accuracy of expression normalization of target genes, particularly for NFs exceeding three (Fu et al., 2013; Zhu et al., 2014; Jia et al., 2019). Therefore, geNorm suggests that the optimal number of NFs should not exceed three (Vandesompele et al., 2002). In addition, the validation results for the RGs suggested that it is essential to use different RGs depending on experimental conditions, and the selection and validation of the RGs for aquatic firefly species was effective in this study.

## Conclusion

In this study, suitable RGs were identified for investigations of ecological adaptation in fireflies. As a case study, this study represents first key step toward establishing a standardized qPCR analysis procedure for firefly species. Overall, α-*tubulin*, *GST*, β-*actin* and β-*tubulin* were effective RGs in five different experimental conditions. As additional genomes and transcriptomes of various firefly species are obtained, the results of this study lay a foundation for subsequent research in genomics and functional genomics. Notably, for other experimental conditions, it will be essential to evaluate the stability of candidate RGs by standard processes.

## Data Availability Statement

All datasets generated for this study are included in the article/Supplementary Material.

## Author Contributions

Q-LZ and X-YD conceived and designed the study. X-JY, H-LZ, Y-YL, H-WL, and Y-HJ performed the experiments. Q-LZ, X-JY, H-LZ, Y-YL, H-WL, and Y-HJ analyzed the data. Q-LZ and X-JY carried out the statistics. Q-LZ, X-JY, H-WL, and Y-HJ drafted the manuscript. L-BL, X-YD, and Q-LZ revised the manuscript. All authors have read, commented on, and approved the manuscript.

## Conflict of Interest

The authors declare that the research was conducted in the absence of any commercial or financial relationships that could be construed as a potential conflict of interest.

## Footnotes

1 http://www.gene-quantification.de/bestkeeper.html

2 https://genorm.cmgg.be/

3 http://moma.dk/normfinder-software

4 https://omictools.com/reffinder-tool

## Supplementary Material

Supplementary Figure 1 Specificity and product length of PCR amplification for ten candidate RGs, including α-*tubulin* **(a)**, β-*tubulin* **(b)**, β-*actin* **(c)**, *EFA* **(d)**, *SDHA* **(e)**, *UBQ* **(f)**, *GST* **(g)**, *GAPDH* **(h)**, *RPS31* **(i)**, *RPL13A* **(j)**.

Supplementary Figure 2 Ct values of ten candidate reference genes among samples for each experimental group. A, different normal tissues; B, different temperature; C, different sex; D, different developmental stages; E, different dose of benzopyrene (mg/L). The line in the box indicates median. The box describes between 25th and 75th percentiles, the maximum and minimum values and were represented by up and low caps.

Supplementary Table 1 Ct values (mean ± SD) of candidate reference genes of *Aquatica leii* under five different experimental groups.

Supplementary Table 2 Expression stability (SD ± [CP]) of candidate reference genes in *Aquatica leii* under different experimental groups calculated by BestKeeper.

Supplementary Table 3 Expression stability (*M*) of candidate reference genes in *Aquatica leii* under different experimental groups calculated by geNorm.

Supplementary Table 4 Expression stability of candidate reference genes in *Aquatica leii* under different experimental groups calculated by NormFinder.

## Abbreviations

BaP: benzo(a)pyrene
CV: coefficient of variation
NFs: normalization factors
PAHs: polycyclic aromatic hydrocarbons
qPCR: quantitative real-time PCR
RGs: reference genes
SD: standard deviation.
