# Supplementary material for: Selection of Reference Genes for Quantitative Real-Time PCR in Aquatica leii (Coleoptera: Lampyridae) Under Five Different Experimental Conditions
Source: Front Physiol. 2020 Oct 6;11:555233. doi: 10.3389/fphys.2020.555233 (PMC7573347; doi:10.3389/fphys.2020.555233)
Supplement: Supplementary Figure 1 — Specificity and product length of PCR amplification for ten candidate RGs, including α-tubulin (a), β-tubulin (b), β-actin (c), EFA (d), SDHA (e), UBQ (f), GST (g), GAPDH (h), RPS31 (i), RPL13A (j). [file Table_1.DOCX]

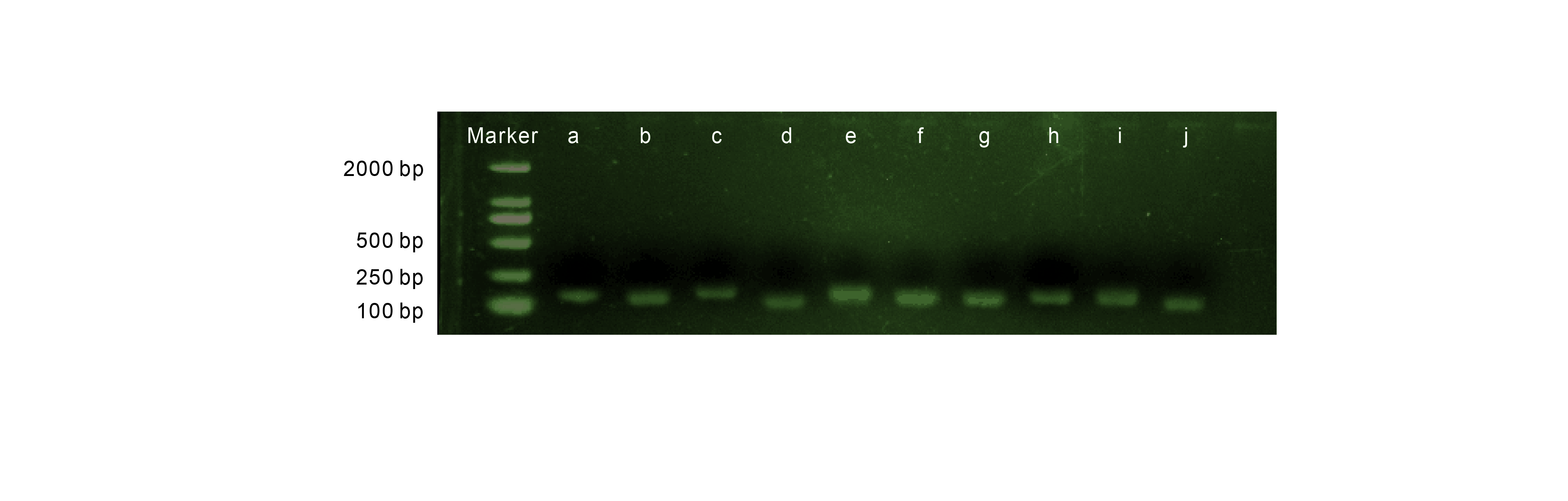


**Supplementary Figure 1** Specificity and product length of PCR amplification for ten candidate RGs, including α-tubulin (a), β-tubulin (b), β-actin (c), EFA (d), SDHA (e), UBQ (f), GST(g), GAPDH (h), RPS31 (i), RPL13A (j).
