# Supplementary material for: Selection of Reference Genes for Quantitative Real-Time PCR in Aquatica leii (Coleoptera: Lampyridae) Under Five Different Experimental Conditions
Source: Front Physiol. 2020 Oct 6;11:555233. doi: 10.3389/fphys.2020.555233 (PMC7573347; doi:10.3389/fphys.2020.555233)
Supplement: Supplementary Figure 2 — Ct values of ten candidate reference genes among samples for each experimental group. A, different normal tissues; B, different temperature; C, different sex; D, different developmental stages; E, different dose of benzopyrene (mg/L). The line in the box indicates median. The box describes between 25th and 75th percentiles, the maximum and minimum values and were represented by up and low caps. [file Table_2.DOCX]

**
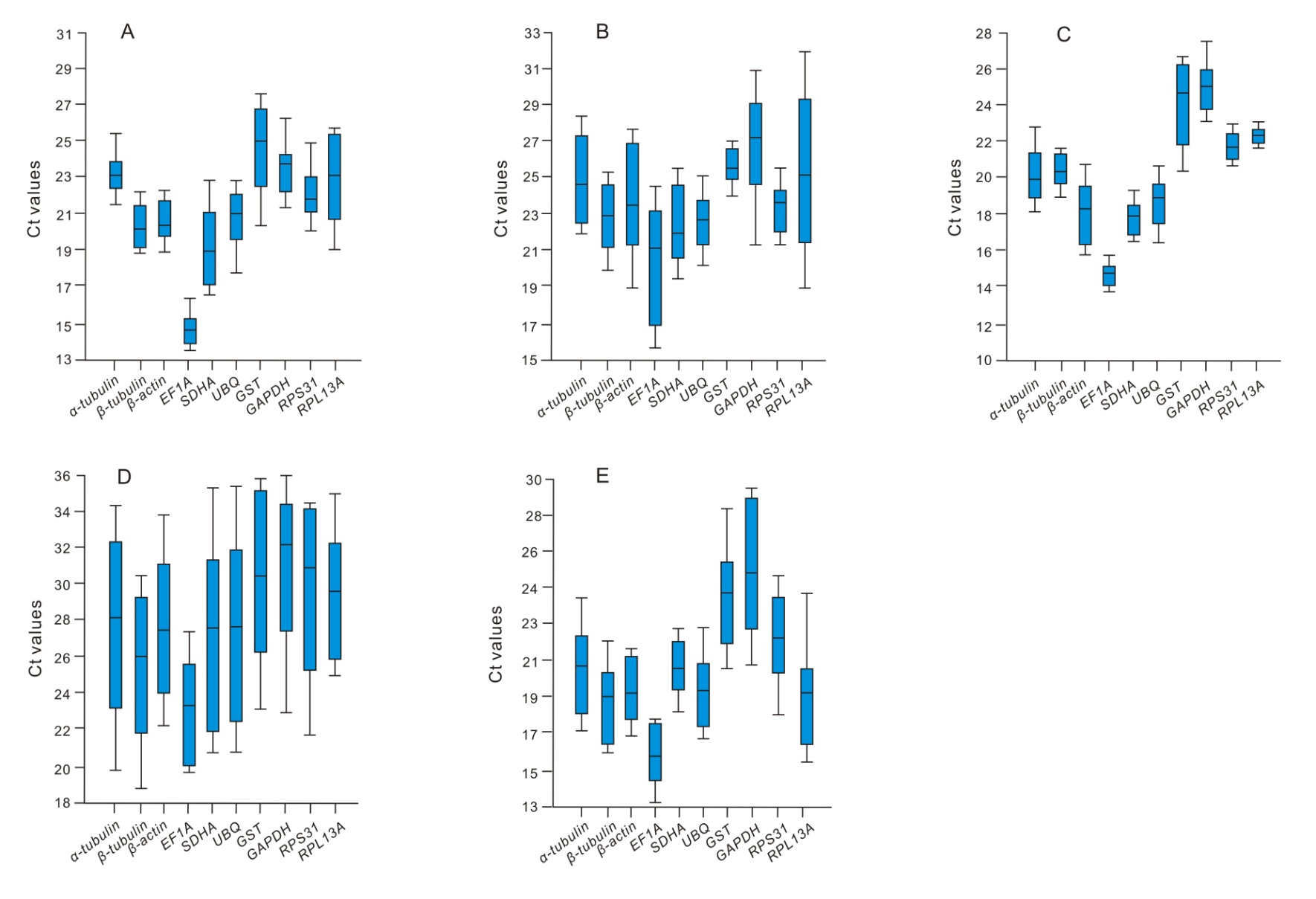
**

**Supplementary Figure 2** **Ct values of ten can****didate reference genes among samples for each experimental condition**. A, different normal tissues; B, different temperature; C, different sex; D, different developmental stages; E, different dose of benzopyrene (mg/L). The line in the box indicates median. The box describes between 25th and 75th percentiles, the maximum and minimum values and were represented by up and low caps.
