# Supplementary material for: Selection of Reference Genes for Quantitative Real-Time PCR in Aquatica leii (Coleoptera: Lampyridae) Under Five Different Experimental Conditions
Source: Front Physiol. 2020 Oct 6;11:555233. doi: 10.3389/fphys.2020.555233 (PMC7573347; doi:10.3389/fphys.2020.555233)
Supplement: Supplementary Table 1 — Ct values (mean ± SD) of candidate reference genes of Aquatica leii under five different experimental groups. [file Table_3.DOCX]

**Supplementary Table 1** Ct values (mean ± SD) of candidate reference genes of *Aquatica leii* under different treatments.

| Gene | Tissue | Temperature  (°C) | Sex | Developmental stages | Different dose of benzopyrene (mg/L) |
| --- | --- | --- | --- | --- | --- |
| *α-tubulin* | 22.17±1.74 | 24.92±2.15 | 21.23±1.34 | 26.49±4.02 | 21.37±2.92 |
| *β-tubulin* | 19.71±1.15 | 22.27±2.13 | 20.04±0.86 | 25.47±3.81 | 19.34±2.47 |
| *β-actin* | 19.37±1.43 | 22.63±2.15 | 18.34±1.47 | 27.72±3.35 | 18.72±2.38 |
| *EF1A* | 15.54±0.58 | 19.24±2.67 | 14.88±0.51 | 23.59±2.86 | 15.73±2.32 |
| *SDHA* | 19.71±2.24 | 21.78±2.47 | 17.96±0.93 | 27.16±4.59 | 19.92±2.06 |
| *UBQ* | 19.88±1.12 | 22.34±1.82 | 18.83±1.21 | 29.13±4.37 | 19.56±2.41 |
| *GST* | 23.96±2.16 | 25.83±1.56 | 24.16±2.31 | 30.69±4.27 | 24.02±2.78 |
| *GAPDH* | 24.82±1.68 | 26.78±2.33 | 24.91±1.41 | 32.84±4.93 | 23.79±3.62 |
| *RPS31* | 22.87±1.31 | 23.31±1.68 | 20.97±0.89 | 31.86±4.35 | 21.45±2.73 |
| *RPL13A* | 21.35±2.16 | 24.35±2.96 | 22.73±0.36 | 31.52±3.13 | 20.47±2.74 |

Tissues: head, thorax, abdomen , antenna of adult; Temperature: adults exposed to 15, 20, 25, 30, and 30 °C for 2 h, respectively; Sex: female and male adults; Stage of development: eggs, larvae, pupae, and adults; Benzo pyrene treatment at different concentrations: 0.1 mg/L, 0.01 mg/L, 0.001 mg/L, and control. The same below.
