# Supplementary material for: Selection of Reference Genes for Quantitative Real-Time PCR in Aquatica leii (Coleoptera: Lampyridae) Under Five Different Experimental Conditions
Source: Front Physiol. 2020 Oct 6;11:555233. doi: 10.3389/fphys.2020.555233 (PMC7573347; doi:10.3389/fphys.2020.555233)
Supplement: Supplementary Table 2 — Expression stability (SD ± [CP]) of candidate reference genes in Aquatica leii under different experimental groups calculated by BestKeeper. [file Table_4.DOCX]

**Supplementary Table 2** Expression stability (SD ± [CP]) of candidate reference genes in *Aquatica leii* under different treatments calculated by BestKeeper.

| Gene | Tissue | Temperature | Sex | Developmental stages | Different dose of benzopyrene |
| --- | --- | --- | --- | --- | --- |
| *α-tubulin* | 0.32 | 1.04 | 0.57 | 3.72 | 0.12 |
| *β-tubulin* | 0.57 | 1.32 | 0.28 | 2.94 | 0.37 |
| *β-actin* | 1.60 | 1.84 | 0.38 | 3.84 | 0.58 |
| *EF1A* | 1.17 | 1.56 | 0.32 | 3.06 | 0.27 |
| *SDHA* | 1.43 | 1.48 | 0.88 | 2.86 | 0.78 |
| *UBQ* | 1.29 | 1.77 | 1.12 | 3.89 | 0.61 |
| *GST* | 1.15 | 0.87 | 0.62 | 3.27 | 0.45 |
| *GAPDH* | 1.54 | 1.63 | 1.26 | 2.74 | 0.84 |
| *RPS31* | 1.25 | 1.99 | 0.94 | 3.43 | 0.33 |
| *RPL13A* | 1.51 | 1.91 | 0.46 | 3.59 | 0.92 |
