# Supplementary material for: Selection of Reference Genes for Quantitative Real-Time PCR in Aquatica leii (Coleoptera: Lampyridae) Under Five Different Experimental Conditions
Source: Front Physiol. 2020 Oct 6;11:555233. doi: 10.3389/fphys.2020.555233 (PMC7573347; doi:10.3389/fphys.2020.555233)
Supplement: Supplementary Table 3 — Expression stability (M) of candidate reference genes in Aquatica leii under different experimental groups calculated by geNorm. [file Table_5.DOCX]

**Supplementary Table 3** Expression stability (*M*) of candidate reference genes in *Aquatica leii* under different treatments calculated by geNorm.

| Gene | Tissue | Temperature | Sex | Developmental stages | Different dose of benzopyrene |
| --- | --- | --- | --- | --- | --- |
| *α-tubulin* | 0.57 | 2.41 | 1.22 | 3.47 | 0.24 |
| *β-tubulin* | 0.57 | 2.59 | 0.99 | 2.53 | 0.44 |
| *β-actin* | 1.73 | 2.27 | 0.62 | 3.36 | 0.88 |
| *EF1A* | 1.48 | 2.66 | 0.86 | 2.96 | 0.24 |
| *SDHA* | 1.26 | 2.14 | 1.46 | 3.68 | 0.47 |
| *UBQ* | 1.42 | 2.74 | 1.78 | 4.23 | 0.83 |
| *GST* | 1.18 | 1.84 | 1.64 | 2.53 | 0.61 |
| *GAPDH* | 1.87 | 1.84 | 1.66 | 3.89 | 0.79 |
| *RPS31* | 1.03 | 2.92 | 0.62 | 3.94 | 0.35 |
| *RPL13A* | 1.66 | 2.88 | 1.55 | 4.42 | 0.96 |
