# Supplementary material for: Selection of Reference Genes for Quantitative Real-Time PCR in Aquatica leii (Coleoptera: Lampyridae) Under Five Different Experimental Conditions
Source: Front Physiol. 2020 Oct 6;11:555233. doi: 10.3389/fphys.2020.555233 (PMC7573347; doi:10.3389/fphys.2020.555233)
Supplement: Supplementary Table 4 — Expression stability of candidate reference genes in Aquatica leii under different experimental groups calculated by NormFinder. [file Table_6.DOCX]

**Supplementary Table 4** Expression stability of candidate reference genes in *Aquatica leii* under different treatments calculated by NormFinder.

| Gene | Tissue | Temperature | Sex | Developmental stages | Different dose of benzopyrene |
| --- | --- | --- | --- | --- | --- |
| *α-tubulin* | 0.17 | 1.56 | 0.52 | 2.49 | 0.27 |
| *β-tubulin* | 0.34 | 1.58 | 0.24 | 1.76 | 0.33 |
| *β-actin* | 1.36 | 1.43 | 0.11 | 2.38 | 0.78 |
| *EF1A* | 0.93 | 1.70 | 0.20 | 2.15 | 0.29 |
| *SDHA* | 0.76 | 1.28 | 0.71 | 2.69 | 0.38 |
| *UBQ* | 0.72 | 1.66 | 1.16 | 3.26 | 0.87 |
| *GST* | 0.65 | 0.96 | 1.04 | 2.34 | 0.62 |
| *GAPDH* | 1.43 | 1.24 | 0.76 | 2.73 | 0.61 |
| *RPS31* | 0.48 | 1.92 | 0.28 | 2.95 | 0.14 |
| *RPL13A* | 1.14 | 1.74 | 0.99 | 3.54 | 0.89 |
